# A Novel Phenanthridionone Based Scaffold As a Potential Inhibitor of the BRD2 Bromodomain: Crystal Structure of the Complex

**DOI:** 10.1371/journal.pone.0156344

**Published:** 2016-05-31

**Authors:** Shailesh Tripathi, Shruti Mathur, Prashant Deshmukh, Ramu Manjula, Balasundaram Padmanabhan

**Affiliations:** Department of Biophysics, National Institute of Mental Health and Neurosciences (NIMHANS), Hosur Road, Bangalore, 560029, India; NCI-Frederick, UNITED STATES

## Abstract

Bromodomain containing proteins recognize the level of histone acetylation and regulate epigenetically controlled processes like gene transcription and chromatin modification. The BET (bromodomain and extra-terminal) family proteins, which are transcriptional co-regulators, have been implicated in the pathogenesis of cancer, neurodegenerative disorders, and defects in embryonic stem cell differentiation. Inhibitors selectively targeting the BET bromodomains can pave the path for new drug discovery against several forms of major diseases. By a rational structure-based approach, we have identified a new inhibitor (NSC127133) of the second bromodomain (BD2) of the BET family protein BRD2 using the NCI Diversity Set III library. A high-resolution crystal structure of the BRD2-BD2 in complex with this compound and in *apo*- form is refined to 0.91 and 0.94 Å, respectively. The compound, which is a phenanthridinone derivative, binds well to the acetyl-lysine binding pocket of BD2 and displays significant hydrophobic and hydrophilic interactions. Moreover, the atomic resolution data obtained in this study allowed us to visualize certain structural features of BD2 which remained unobserved so far. We propose that the discovered compound may be a potential molecule to develop a new library for inhibiting the BRD2-BD2 function.

## Introduction

The nucleosome is a primary component of chromatin, packing 147 base pair length of DNA around a histone octamer, leading to an organized and compact chromatin [1]. The post-translational modifications such as acetylation of lysine residues (Kac) on the histone tails and the recognition of acetylated-histone tails are a typical hallmark of transcription activation [2]. Bromodomains are known to be the epigenetic readers. They selectively recognize acetylated lysine residues on the histone tails, a key event in reading the post-translational modifications [3]. The bromodomain, which consists of about 110 amino acids, is present in several chromatin associated factors including nuclear histone acetyl transferases (HATs), chromatin associated factors and bromodomain and extra terminal (BET) domain family nuclear proteins [4].

The BET family proteins (BRD2, BRD3, BRD4, and BRDT) contain two tandem bromodomains and an extra-terminal domain at the C-terminal. The BET family proteins generally recognize acetylated histones H3 and H4 ([5] and references therein); however, BRD2 preferentially recognize mono/di-acetylated histone tail of H4 [6,7]. The tertiary structure of all bromodomains contains a left-handed α-helical bundle formed by four α-helices (α_Z_, α_A_, α_B_, and α_C_). The α-helical bundle along with flanking ZA and BC loops create a deep hydrophobic cavity, which recognizes a specific sequence of acetylated-histone tails [8].

As BRD2 and BRD4 are known to remain associated with mitotic chromatin, it has been suggested that they may have a role in preserving transcriptional memory in cell division processes [9]. The biological function of the BET family proteins has recently been reviewed [10]. The BET family proteins are involved in transcriptional co-repression as well as transcriptional co-activation. For example, they co-repress the transcriptional activity such as insulin transcription [11], PPAR-γ –controlled adipogenic differentiation in adipose tissue [12] and GATA-controlled haematopoietic differentiation [13]. Similarly, they co-activate the transcription activity like the activation of the genes responsible for cell cycle progression [14] and NF-κB–controlled synthesis of pro-inflammatory cytokines [15]. It has been reported that BRD2 and BRD4 involve in initiating transcription elongation, by mediating in positive transcription elongation factor b (P-TEFb) assembly [16]. The BET proteins recruit P-TEFb to nucleosomes by directly interacting with it.

Dysfunction of BET proteins has been implicated in several forms of cancers, obesity, inflammation and neurodegenerative diseases [17–20]. Inhibition of BET bromodomains is envisioned to help in evading several forms of cancers, by halting the expression of genes important for tumor growth [5]. Recently, several selective and highly potent pan-BET inhibitors have been extensively pursued by the scientific community, demonstrating their potential anti-tumor activity in cell [21–25]. The five BET family inhibitors, namely RVX-208, I-BET 762, OTX 015, CPI-0610 and TEN-010 were registered for active clinical trials, for investigating the BET proteins against various diseases [22]. These inhibitors have been shown to exhibit significant anticancer activity in NUT midline carcinoma, multiple myeloma, acute myeloid leukemia, lung cancer, pancreatic ductal adenocarcinoma and glioblastoma [5,26]. These inhibitors engage the acetylated lysine binding site, and most of them make hydrogen bonds to the conserved binding site asparagine.

To find structurally diverse potential molecules, we carried out structure based approaches of virtual screening and X-ray crystallography to obtain novel inhibitors of BRD2 using the NCI Diversity Set III library. Here, we discuss a newly identified small molecule inhibitor (NSC127133; hereafter, L10), a phenanthridinone derivative, of the second bromodomain (BD2) of BRD2.

## Materials and Methods

### Virtual Screening and Docking

The crystal structure of the second bromodomain of BRD2 in complex with an inhibitor, JQ1 (BRD2-BD2: aa: 344–455; PDB Id: 3ONI) [27] was used as a model for docking studies. The ligand, JQ1 and the water molecules were removed from this structure. The target protein structure was converted into the required PDBQT format from the PDB format using PyRx [28]. The NCI Diversity Set III, which contains 1700 compounds, downloaded from the NCI database (http://dtp.nci.nih.gov/), was used for virtual screening. For compounds, generation of 3D structures, the addition of polar hydrogens and partial charges to ligand atoms and their subsequent conversion into the required PDBQT format were accomplished by OpenBabel 2.3.1. [29]. All appropriate bonds were allowed to have a free rotation. The parameters for rotational bonds and Gasteiger charges were assigned to each compound.

All computational work was performed on the Intel Core i5 processor and 4 GB of RAM running on the Ubuntu 12.04 operating system. Initially, AutoGrid and AutoDock 4.2.5 [30] were used to perform the docking of known JQ1 inhibitor complex (PDB Id: 3ONI) to validate the docking procedures prior to performing virtual screening against the NCI Diversity Set III database. Cues were also taken from these docking runs to optimize the grid size and docking parameters. The docked JQ1 compound structure was closely identical (RMSD: 0.50 Å) to its crystal structure (S1 Fig). The optimized grid parameters were subsequently used for dockings against the library compounds using the program AutoDock Vina 1.1.2 [31]. The grid size was set to 25Å × 25Å × 25Å comprising the Kac-binding site in which ligands were allowed to move freely. Exhaustiveness was set to 50 with all other parameters set to default values.

The docked compounds were sorted based on their binding energy values, and the top-ranked compounds were subsequently inspected visually on the graphics for good chemical geometry and docking (S1 Table). The criteria for the hydrogen bond formation was set as H-bond acceptor distance less than 3.5 Å and the H-bond donor-H-bond-acceptor angle larger than 120° in the protein-ligand complexes [32]. For molecular visualization, docking poses generated by AutoDock Vina were directly loaded into PyMol through PyMol Autodock/Vina Plugin. Pictures of the modeled protein-ligands complex were produced by PyMol [33].

### Docking Energy Analysis

Another robust program, DSX (DrugScore eXtented) [34] was used to further confirm the docking results of the protein-ligand complexes. DSX was used to estimate the binding energy of the ligands bound to the BD2 structure. It uses a knowledge-based scoring function based on the DrugScore formalism. It also allows visualization of per-atom score contributions. The protein-ligand with the larger negative score has a theoretical higher affinity.

### Ligand Efficiency Analysis

The ligand efficiency (LE) is an index of how 'efficiently' the non-hydrogen atoms contribute to the overall interaction of the molecule. For this reason, the absolute value of the energy (score) is not always the right criterion for selecting hits. The 'ligand efficiency’ (LE) is defined by LE = -ΔG/HA, where –ΔG is the free energy of binding and HA is the number of non-hydrogen atoms of the ligand [35]. The compounds showing LE > 0.29 kcal^-1^ mol^-1^HA^-1^ were accepted as good starting hits for lead optimization. The MGL tools were used to calculate the LE of ligands.

### Cloning, protein production, and purification

The sequence corresponding to the second bromodomain of the human BRD2 protein (residues 348-455aa), containing an insertion encoding for a hexa-histidine tag in the N-terminal region was cloned into a pET28a vector. BL21 Star (DE3) cells were transformed with this vector. The cells were initially grown at 37°C for 3–4 hours. Induction was performed using IPTG (0.5 mM final concentration), and the cells were further grown for 5 hrs at 37°C. The cells were harvested and re-suspended in lysis buffer (50 mM Tris-HCl pH 7.5, 200 mM NaCl). The cells were lysed (sonication) and centrifuged (22,000 rpm for 45 minutes at 4°C) to remove the cell debris. The supernatant was loaded on to a Ni-NTA column for Ni-affinity purification. The column was first equilibrated with a buffer containing 50 mM Tris-HCl pH 7.5, 200 mM NaCl, 10 mM imidazole. The His-tag BRD2-BD2 protein was eluted with the same buffer, but containing 150 mM imidazole. Imidazole was removed by multiple dilution and concentration steps using a buffer containing 50 mM Tris-HCl pH 7.5 and 200 mM NaCl. The protein was concentrated, and the His-tag was removed by thrombin (Sigma) digestion (overnight incubation at 4°C). The undigested protein and the cleaved His-tag were removed by performing second Ni-NTA purification. Finally, size-exclusion chromatography was performed to obtain high purity BD2 protein. Buffer exchange was performed by multiple dilution and concentration steps to obtain finally 10 mg/ml protein concentration, in a buffer containing 50 mM Tris-HCl pH 7.5 and 50 mM NaCl.

### Co-crystallization of potential inhibitor compounds

Prior to crystallization, the predicted 10 compounds were separately mixed with the BRD2-BD2 protein solution to obtain 5 mg/ml final protein concentration, and saturated compound concentration in 5% DMSO, 50 mM Tris-HCl pH 7.5 and 50 mM NaCl, and incubated overnight at 4°C. Crystallization was performed by sitting-drop vapor diffusion method, by mixing 1 μl of each protein-ligand complexes with 1 μl of reservoir solution (30% PEG MME 2000, 50 mM Tris-HCl pH 7.5, 50 mM NaCl, 20% glycerol) and equilibrated with 1 ml of reservoir solution at 20°C. For the *apo-*form, 1 μl of BD2 protein was used instead of protein-ligand complexes. The crystals were observed overnight and grew in the form of thin plates (20–30 μm thick) within a week.

### X-ray data collection and processing

X-ray data was collected on the beamline BM-14U at the European synchrotron radiation facility (ESRF, Grenoble, France). The data sets were collected at 100 K, using the MarCCD detector. The crystal-to-detector was set to 105 mm. X-ray beam of wavelength 0.729 Å with an exposure time of 0.1 seconds was used for each frame. The oscillation range per image was 1.0°, with no overlap between two contiguous images. The diffraction data, corresponding to the *apo-*form as well as for 10 protein-ligand complexes, were integrated and reduced using MOSFLM [36]. The reduced data was scaled using SCALA [37]. The crystals of *apo-*form and complexes of BRD2-BD2 belong to the primitive orthorhombic space group *P*22_1_2_1_. Data statistics are shown in Table 1 for the *apo-*form and the *BRD2-BD2 –L10* complex.

**Table 1:** Data reduction and refinement statistics of the *apo-*form of BRD2-BD2 and *BD2-L10* structures.

	BD2 *Apo-*form	*BD2-L10* complex
Data collection		
Space group	*P*22_1_2_1_	*P*22_1_2_1_
Cell dimensions, a b c (Å)	32.06 52.46 71.37	32.08 52.46 71.37
Wavelength (Å)	0.7293	0.7293
Resolution (Å)^#^	40.0–0.94 (0.99–0.94)	40.0–0.91 (0.96–0.91)
Unique reflections	78817 (11418)	86054 (12530)
R_meas_ ^††^	0.1 (1.948)	0.079 (1.541)
	(1.387)*	(1.014)**
⟨I/σ(I)⟩	9.5 (1.0)	11.6 (0.9)
	(1.6)*	(1.6)**
Completeness (%)	99.7 (99.7)	99.4 (100)
Redundancy	8.0 (7.9)	4.4 (4.1)
CC_1/2_	0.999 (0.309)	0.999 (0.315)
	(0.539)*	(0.541)**
Wilson plot B-factor (Å^2^)	8.1	7.0
Refinement		
Resolution (Å)	20.0–0.94	20.0–0.91
No. of reflections	74948	81790
^ǂ^R_work_ / ^+^R_free_	13.14 / 14.85	12.5 / 14.3
Total no. of atoms	1240	1333
Protein atoms	1003	1073
Water molecules	164	213
CL^ˉ^ ions	1	2
Glycerol molecules	5	2
PEG molecules	2	-
Ligand (L10)	-	1
Average B-factor (Å^2^)	13.46	12.23
RMSD Bonds (Å)	0.019	0.020
RMSD Angles (°)	1.98	1.98

^#^Numbers in parentheses are values in the highest resolution shell.

††*R*_*meas*_ = ∑_*hkl*_{*N*(*hkl*)/[*N*(*hkl*) − 1]}^1/2^ × ∑_*i*_|*I*_*i*_(*hkl*) − 〈*I*(*hkl*)〉|/∑_*hkl*_ ∑_*i*_ *I*_*i*_(*hkl*), where N(hkl) is the multiplicity of I(hkl) and 〈*I*(*hkl*)〉 is the mean intensity of that reflection.

ǂR_*work*_ = ∑_*hkl*_||*F*_*obs*_| − |*F*_*calc*_||/∑_*hkl*_|*F*_*obs*_|, where |*F*_*obs*_| and |*F*_*calc*_| are the observed and calculated structure-factor amplitudes, respectively.

^+^*R*_*free*_ was calculated with 5.0% of reflections in the test set.

* Values in parentheses are for the highest resolution shell (1.02–0.97 Å), if the resolution is truncated to 0.97 Å for the *apo-BD2* data.

** Values in parentheses are for the highest resolution shell (0.99–0.94 Å), if the resolution is truncated to 0.94 Å for the *BD2-L10* data.

### Structure determination and refinement

The crystal structure of the *apo-*form and 10 complexes of BRD2-BD2 were solved by the molecular replacement method using *Phaser-MR* as implemented in the *PHENIX* package [38], with the BRD2-BD2 structure (PDB Id: 2E3K) [7] as a model. The 2|Fo|-|Fc| map unambiguously revealed the structure of BD2. The difference Fourier map clearly revealed the electron density for the compound #10 (L10) of the *BD2-L10* complex; however, the electron density for the other nine compounds was absent in their corresponding BD2 complexes (not shown). The energy minimized coordinates and crystal information file (CIF) for L10 were produced using Jligand [39]. The initial refinement of the structures was performed using the module *Phenix*.*refine* of the *PHENIX* package [38]. In the later stage of the refinement cycles, the refinement of the *apo-* form and the protein complex were performed using *REFMAC5* [40], incorporated in the *CCP4* package [37]. During final stages of refinement of the structures, hydrogen atoms were included in their riding positions, and they were used for geometry gradient calculation and in structure factor calculation. For the *apo-*form, the final refined model in the asymmetric unit contains 111 residues, 2 PEG molecules, 1 Clˉ ion, and 164 water molecules, with a final *R*_work_ of 13.13% and an *R*_free_ of 14.85% at 0.94 Å resolution. For the *BD2-L10* complex, the final refined model in the asymmetric unit contains 115 residues, 2 Clˉ ions, and 213 water molecules, with a final *R*_work_ of 12.5% and an *R*_free_ of 14.3% at 0.91 Å resolution. The stereochemistry of the both the structures is good, as assessed with *MOLPROBITY* [41]. The Ramachandran plot analysis of the structures revealed that 100% of all residues were in the allowed region. The refinement statistics are summarized in Table 1. The structural coordinates of the *apo-*form and L10 complex of BD2 have been deposited in the RCSB (PDB code: 5IBN and 5IG6, respectively).

### Surface Plasmon Resonance binding assay

The binding study of L10 towards BRD2-BD2 was performed using Biacore T200 (GE Healthcare). BRD2-BD2 (25 μg/ml in 20 mM HEPES pH 7.0) was immobilized on the dextran matrix surface of the gold sensor chip CM5 (Biacore, GE Healthcare) using standard amine coupling. Seven different concentrations of L10 (460, 230, 92, 57.5, 28.75, 14.38 and 7.18 μM) were prepared in a buffer containing 1x HBS-P+ (10 mM HEPES pH 7.4, 150 mM NaCl, 0.05% surfactant P20) and 5% DMSO. The same buffer was used as the running buffer. These seven L10 concentration were run with a flow rate of 30 μl/min over the immobilized BD2, to generate reference corrected association and dissociation curves. The association phase of L10 was followed for 30 seconds, before allowing to dissociate in the running buffer during the next 150 seconds. The surface regeneration was performed using glycine-HCl pH 2.0. Response at equilibrium state (Req) was obtained at different L10 concentrations and fitted using single-site binding equation (Biacore^TM^ assay handbook). The dissociation constant K_D_ was estimated using the fitted curve. All the calculations were performed using Biacore T200 BIAevaluation software (GE Healthcare).

## Results and Discussion

### *In-silico* studies

The AutoDock Vina program [31] was used to obtain hit compounds from the NCI Diversity Set III containing 1700 compounds. The virtual screening results were sorted based on the predicted binding free energies (ΔG_vina_). The PyMol program plugged with AudoDock Vina was used to visually check the predicted binding conformations for the selected conformations from the sorted list. Based on the sorted free energy (binding) values and visual inspection of the first 100 compounds carefully, 10 compounds were predicted to bind well to the Kac-binding pocket of BRD2-BD2. Ligand efficiency (LE) is another useful parameter to check the efficiency of ligand binding [35]. The value of LE ≥ 0.29 is an acceptable value for hit-to-lead compounds. The predicted 10 compounds possess LE greater than 0.29 (S1 Table), and subsequently, these compounds were subjected to co-crystal structure analysis.

### Crystal structure of BRD2-BD2 in complex with the compound NSC127133

**Fig 1:**
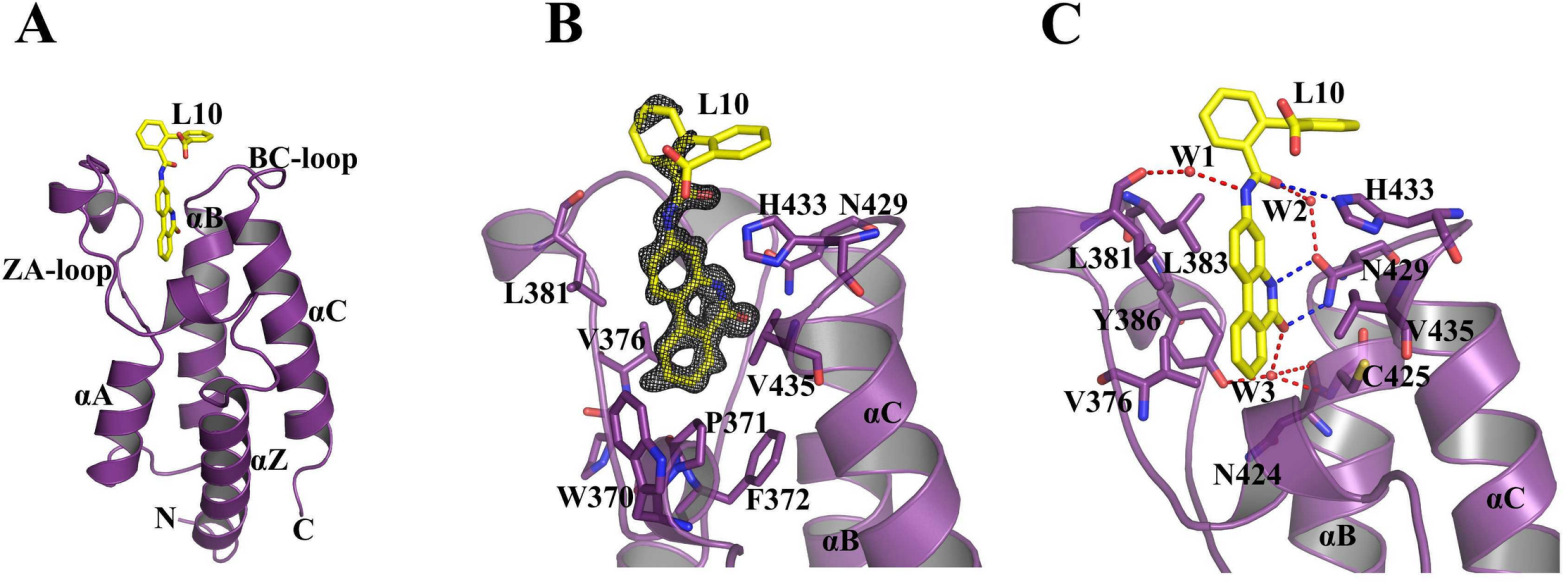
The structure of BRD2-BD2 in complex with L10. **(A)** The tertiary structure of the BRD2-BD2 complex with the compound L10 bound to the active site. (**B) |**2Fo|-|Fc| map of L10 contoured at 1.0σ in the binding pocket of BD2. **(C)** A close-up view of *BD2-L10* interactions. The direct hydrogen bonds are indicated by blue (L10 to H433 and N429) and water mediated ones (through W1, W2, and W3) are indicated by red dotted lines. The water molecule W3 is involved in a three-way hydrogen bond to Y386-OH, N424-O, and C425-NH in addition to the interaction with L10-O9. The water molecule W1 bridges L10-N12 and L381-O. The water molecule W2 forms a similar bridge between L10-O29 and N429-OD1. The ligand and interacting residues are shown as sticks. Water molecules are shown as spheres. The hydrogen bonds are shown as broken lines.

The high-resolution X-ray diffraction data for co-crystals corresponding to the 10 compounds (S1 Table) were used for structure determination (unpublished results). Among them, the L10 complex yielded unambiguous electron density for the compound L10 (NSC127133) at the BD2 binding site (Fig 1). The crystal structure of L10 possesses significant similarity to the one observed in the docking study (RMSD: 0.723 Å) (S2 Fig). The *BD2-L10* crystals diffracted to an atomic resolution of 0.91 Å resolution in *P*22_1_2_1_ space group, with one molecule per asymmetric unit.

The crystal structure obtained at ultra-high resolution data allowed us to analyze the protein-ligand interactions in a detailed manner, as well as to compare with other known structure of the complexes. As observed in the previously determined BD2 structures [7], the BD2 structure consists of a bundle of four α-helices (α_Z_, α_A_, α_B_, α_C_) (Fig 1A). The long loop ZA is connecting α_Z_ and α_A_ helices while the loop BC connects α_B_ and α_C_ helices. These two loops together are forming a deep hydrophobic cavity. The ligand, L10, like other established inhibitors, is occupying the entire binding pocket of BD2 (Fig 1B).

The L10 compound, which binds in the deep hydrophobic cleft, is surrounded by the amino acids P371, V376, L381, L383, Y386, N429, H433 and V435 (Fig 1C). The phenanthridinone moiety, which sits in the deep cleft, is stabilized by both hydrophobic as well as by several direct and water mediated hydrogen bond interactions. The moiety hydrophobically interacts with P371, V376, L381, L383, and V435. One important feature of L10 binding to BD2 is the direct double hydrogen bonds of phenanthridinone O9 and N9 atoms to the carboxamide side chain of N429 (Fig 1C). Moreover, the O9 atom of L10 is also hydrogen bonded to a conserved water molecule W3 (2.92 Å), which forms a three-way hydrogen bond to the backbone amino and carbonyl group of C425 and N424, respectively, and to the sidechain hydroxyl group of Y386. The carbamoyl moiety, which links the phenanthridinone and phenyl-benzoic acid group, makes direct electrostatic interactions with NE2 of H433. Moreover, water-mediated hydrogen bonds are formed between O29 & N12 of the carbamoyl moiety and N429 & L381, respectively (Fig 1C). The phenyl-benzoic acid moiety protrudes away from the binding site and does not have any interactions with the BD2 protein. It correlates well with the observation of weak electron density corresponding to this region (Fig 1B).

### Binding assay for L10 interaction with BRD2-BD2

**Fig 2:**
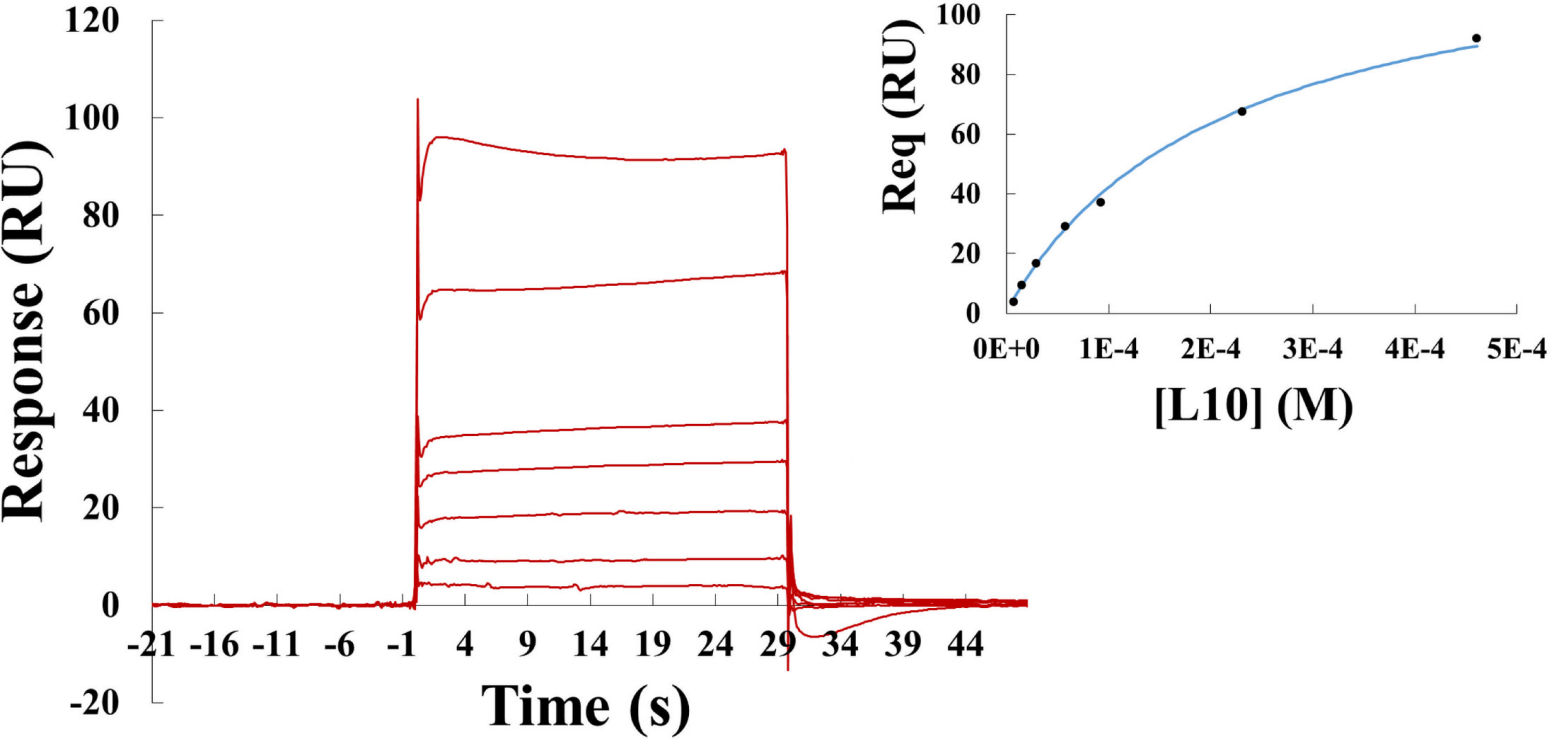
Binding analysis of L10 towards BRD2-BD2. SPR sensorgrams obtained by running different concentrations of L10 over immobilized BRD2-BD2. The figure in the inset shows fitting of data points at equilibrium (Req) for the interaction of different concentrations of L10 to immobilized BD2.

To understand the binding affinity of L10 towards BRD2-BD2, we employed Surface Plasmon Resonance (SPR) analyses using Biacore T200 (GE Healthcare). The amount of immobilized BD2 on the CM5 chip was up to 2420 response units (RUs) for the 420 seconds of coupling time. The assay was performed for seven different concentrations of L10. As speculated, the ligand L10 binds significantly well with BRD2-BD2 and the dissociation constant (K_D_) estimated by globally fitting the equilibrium state response observed at different L10 concentrations is 213.7 μM (Fig 2). As observed in the crystal structure, weak interactions of the terminal phenyl-benzoic acid moiety of L10 may be the possible reason for the moderate affinity of L10 towards BRD2-BD2. The SPR assay together with the crystal structure suggests that the ligand L10 may indeed be a lead molecule to develop high affinity BRD2-BD2 specific inhibitor(s).

### Comparison with the *apo-*form and *BD2-H4K5ac* complex (PDB Id: 2E3K) of BRD2-BD2

**Fig 3:**
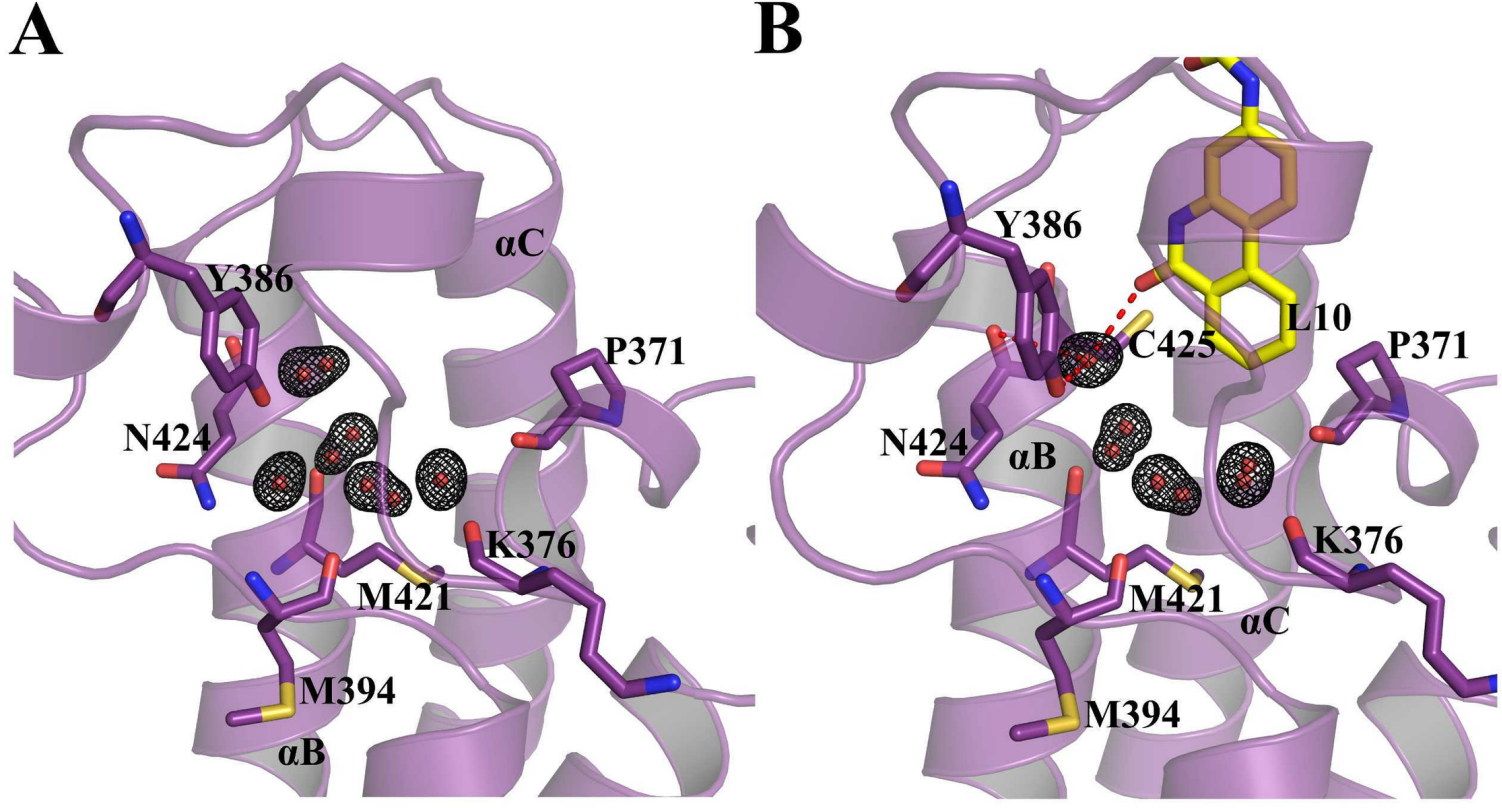
Alternate positions of conserved water molecules. **(A)** Among five conserved water molecules in the *apo-*form binding pocket, three are observed to have alternate positions. (**B)** Three out of four conserved water molecules demonstrate alternate positions in the *BD2-L10* complex. The ligand and selected residues are shown as sticks. Water molecules are shown as spheres. **|**2Fo|-|Fc| map of water molecules contoured at 1.0σ. The hydrogen bonds are shown as broken lines.

**Fig 4:**
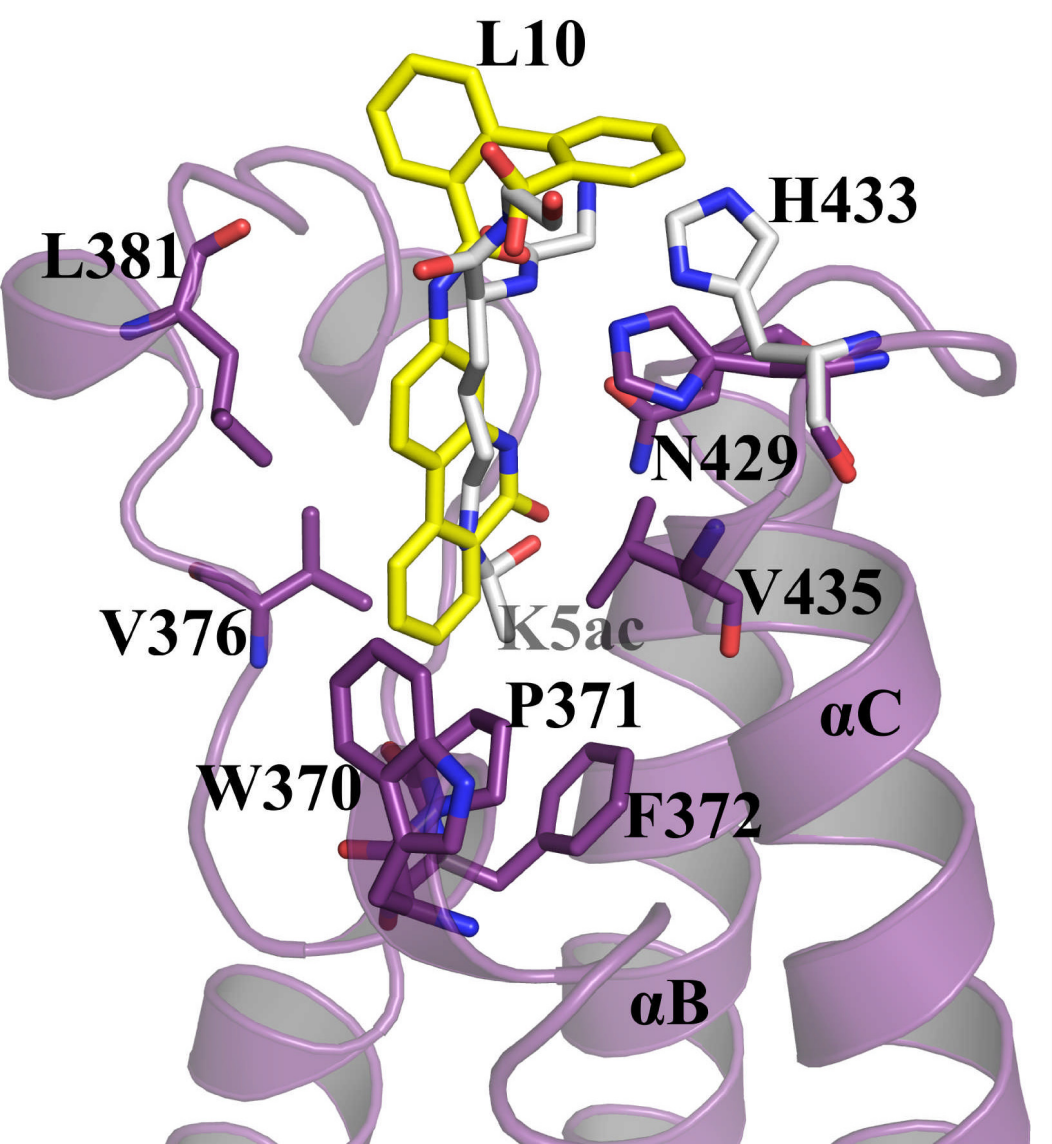
Superposition of *BD2-H4K5ac* (PDB Id: 2E3K) and *BD2-L10* complex. The H4K5ac and L10 are represented in grey and yellow, respectively. The binding site residues corresponding to *BD2-L10* (purple), the ligand L10 (yellow), H433 of *BD2-H4K5ac* (grey) and the *H4K5ac* side-chain (grey) are shown as sticks. H433 is swung in towards the binding pocket in the *BD2-L10* complex.

**Fig 5:**
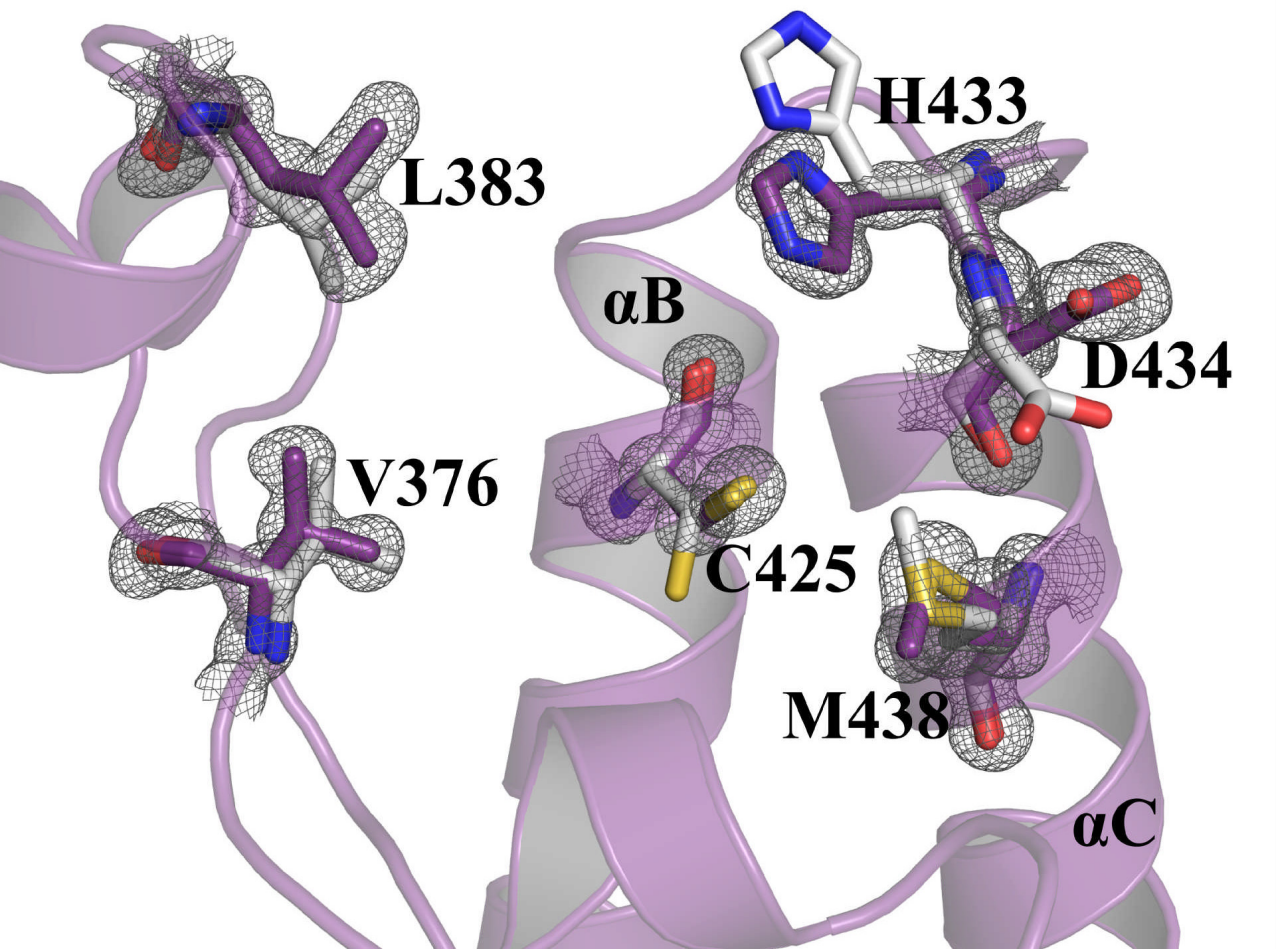
Superposition of BC and ZA loop regions of *BD2-H4K5ac* complex (PDB Id: 2E3K) and *apo-*form. Binding site residues (purple) in the *apo-*form demonstrating conformations different to that observed in the *BD2-H4K5ac* complex structure (grey). The side chain of C425 and M438 are flipped in the *apo-*form compared to the *H4K5ac* structure. The residues near the binding site region, which possess alternate conformations, are shown as sticks. |2Fo|-|Fc| map of the residues contoured at 1.0σ. The hydrogen bonds are shown as broken lines.

**Fig 6:**
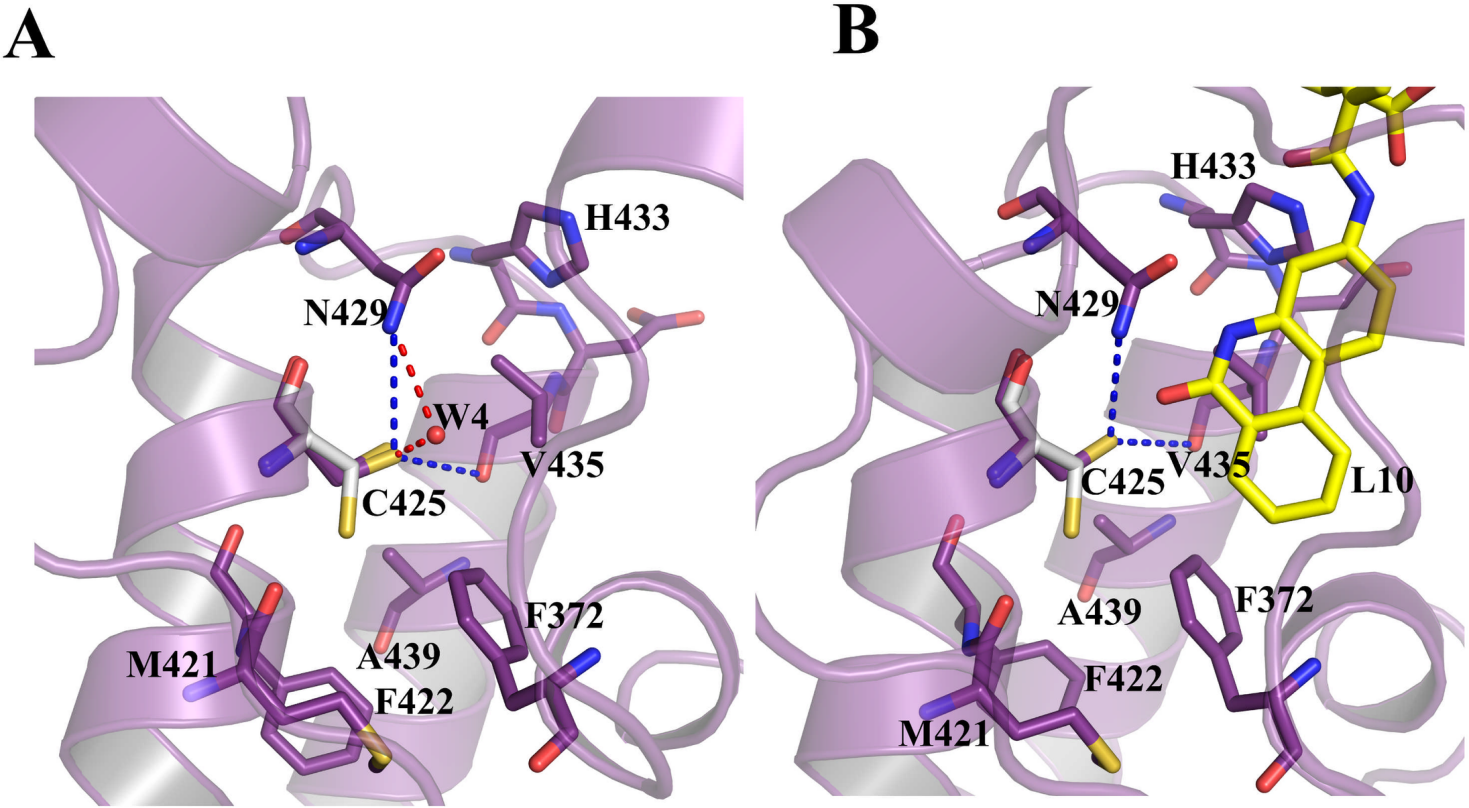
Water molecule hydrogen bonded to C425 and N429 in *apo-*form. **(A)** Direct (blue) and a water mediated (red) hydrogen bond between C425 and N429 in the *apo-*form. **(B)** No water is observed in the same position in the *BD2-L10* complex. Residues corresponding to both *apo-*form and *BD2-L10* are represented in purple. The C425 corresponding to *BD2-H4K5ac* is shown in grey. The ligand and the interacting residues are shown as sticks. Water molecules are shown as spheres. The hydrogen bonds are shown as broken lines.

In both our *apo-* and the *BD2-L10* complex crystal structures, the side chains of several residues are observed to display multiple conformations. This helped us understand the dynamic properties of BD2. Three of the conserved water molecules both in our *apo-BD2* and in the *BD2-L10* complex, are displaying alternate positions (Fig 3). In *apo-BD2*, the alternate positions of water molecules are reciprocated by the alternate conformations of some of the surrounding residues (N424, C425) (S3 Fig). The carbamoyl moiety of Kac is known to hydrogen bond to one of these conserved water molecules [7]. The dynamic nature of these water molecules, as demonstrated by alternate positions, might have a possible role in facilitating the binding of Kac into the binding pocket. Superposition of the *BD2-L10* and *BD2-H4K5ac* [7] complex structures reveals that the binding of phenanthridinone ring moiety of L10 mimics the Kac binding with the BD2 (Fig 4). The difference between our two structures (*apo-* and *BD2-L10*) and *BD2-H4K5ac* complex [7] are quite significant, although differences between *apo-* and *L10* complexes themselves are less. To further analyze structural variations in the binding pocket, we superposed the BC and ZA loop regions (residues 423 to 433 and 380 to 390, respectively) of *H4K5ac*, *apo*- and *BD2-L10* complex structures on to each other. Since the ligand is different in each case, we expected a variant conformation of conserved N429 in each structure but did not observe any. The binding pocket *apo-BD2* residues, displaying a different conformation compared to *H4K5ac* complex, are mostly in the BC loop region (C425, H433, and D434) (Fig 5). The different conformations of residues H433 and D434 arise mainly from interactions with the Kac peptide residues in the *H4K5ac* complex. Compared to the *H4K5ac* complex, the C425 side chain is flipped, both in our *apo-* and *BD2-L10* structures (Fig 6). It forms a direct hydrogen bond to N429-NH_2_ in the *BD2-L10* complex (Fig 6A). In the *apo-*form, C425 contributes an extra water mediated hydrogen bond to N429-NH_2_ (Fig 6B). This water molecule is unique to our *apo-* structure, and it is not observed in the previously reported structures. Alternate position of conserved active site water molecules along with this newly observed water molecule, demonstrate a flexible hydration shell in the binding pocket. Overall, these structural variations together demonstrate a dynamic nature of the binding site, necessary for the activity of the BD2.

### Comparison with known inhibitors

**Fig 7:**
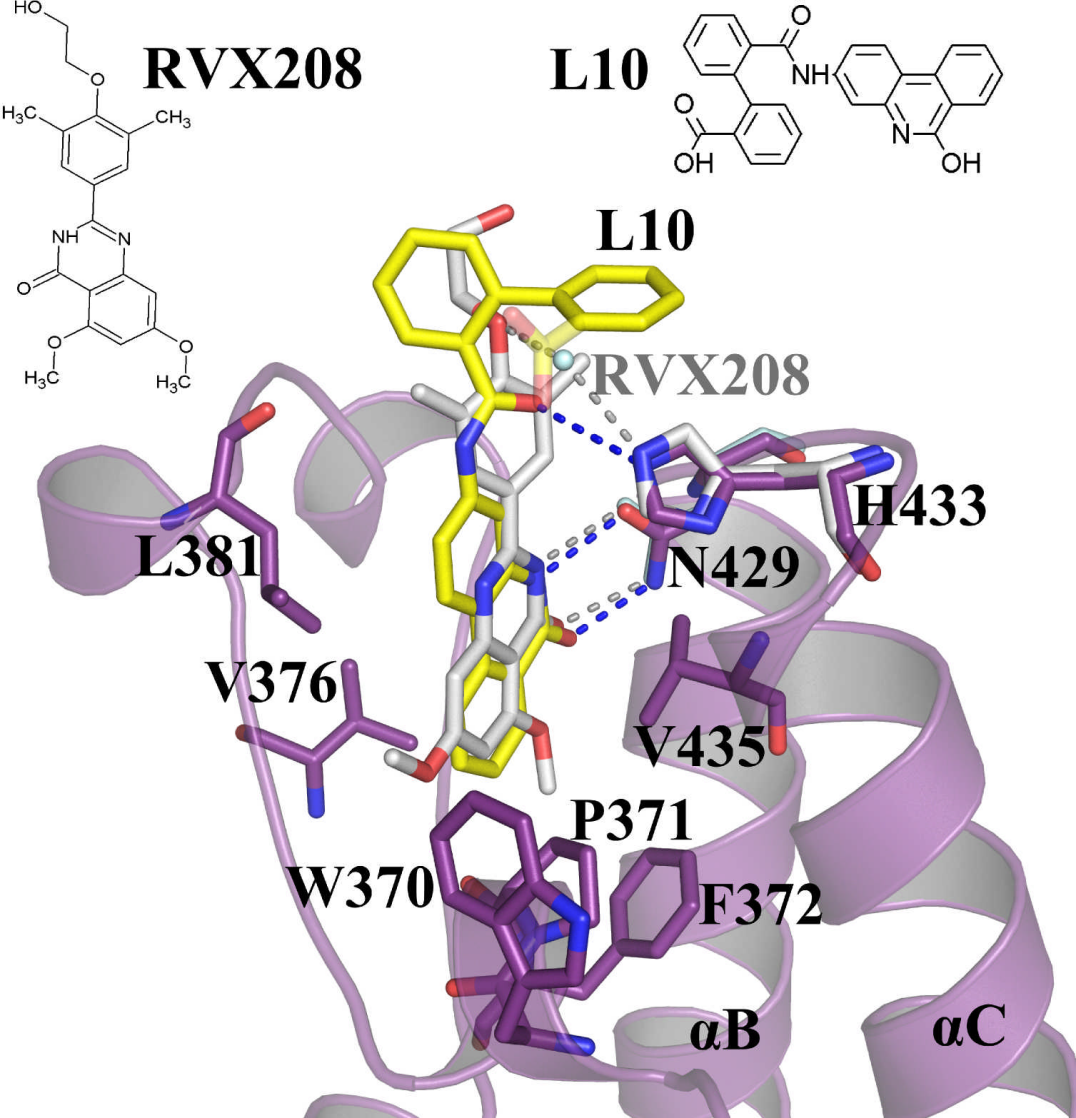
Superposition of the *BD2-RVX-208* and *BD2-L10* complexes. Hydrogen bonding interactions formed by L10 towards N429 and H433 are shown in blue, and by RVX-208 towards N429 and H433 are shown in grey. L10 is shown in yellow. RVX-208 is shown in grey. Residues corresponding to *BD2-L10* and *BD2-RVX-208* are shown in purple and grey, respectively. The ligands and the interacting residues are shown as sticks. The hydrogen bonds are shown as broken lines.

Several crystal structures of the BET family bromodomain–inhibitor complexes have been reported in the literature. However, we have taken two established inhibitor complexes, *BRD2-BD2 –RVX-208* (PDB Id: 4J1P) [42] and *BRD2-BD2 –JQ1* (PDB Id: 3ONI) [27] complexes for comparative studies. To analyze structural deviations with these complexes, RMSD values were calculated by superimposing the structures of *apo-BD2*, *BD2-L10*, *BD2-RVX-208* and *BD2-JQ1* onto each other, for all C^α^ atoms, as well as for the C^α^ atoms in the binding site region covering ZA and BC loops (S2 Table). By comparing *BD2-RVX-208* and *BD2-JQ1*, respectively, *with apo-BD2* and *BD2-L10* complex, *BD2-RVX-208* possesses relatively more structural deviations compared to *BD2-JQ1*, both for all C^α^ atoms and the binding-site region. Superposition of RVX-208 and L10 complexes onto each other, reveals that both the compounds bind to the bromodomain in a similar manner (Fig 7). Strikingly, the hydrogen bonds with N429 in the L10 complex are stronger compared to that found in the RVX-208 complex (Fig 7). The hydrogen bond distances of O9 and N9 atoms of L10 with N429 are 2.84 Å and 2.68 Å, respectively; whereas that observed in RVX-208 are 2.95 Å and 2.84 Å, respectively. The hydrogen bond observed between L10 and H433 is direct, while similar hydrogen bond in RVX-208 complex is water bridged (Fig 7). The inward movement of H433 towards the cleft, which is also observed in *BD2-L10* complex (Fig 4), has been proposed to be the possible reason for tighter affinity of RVX-208 towards BD2 [24].

**Fig 8:**
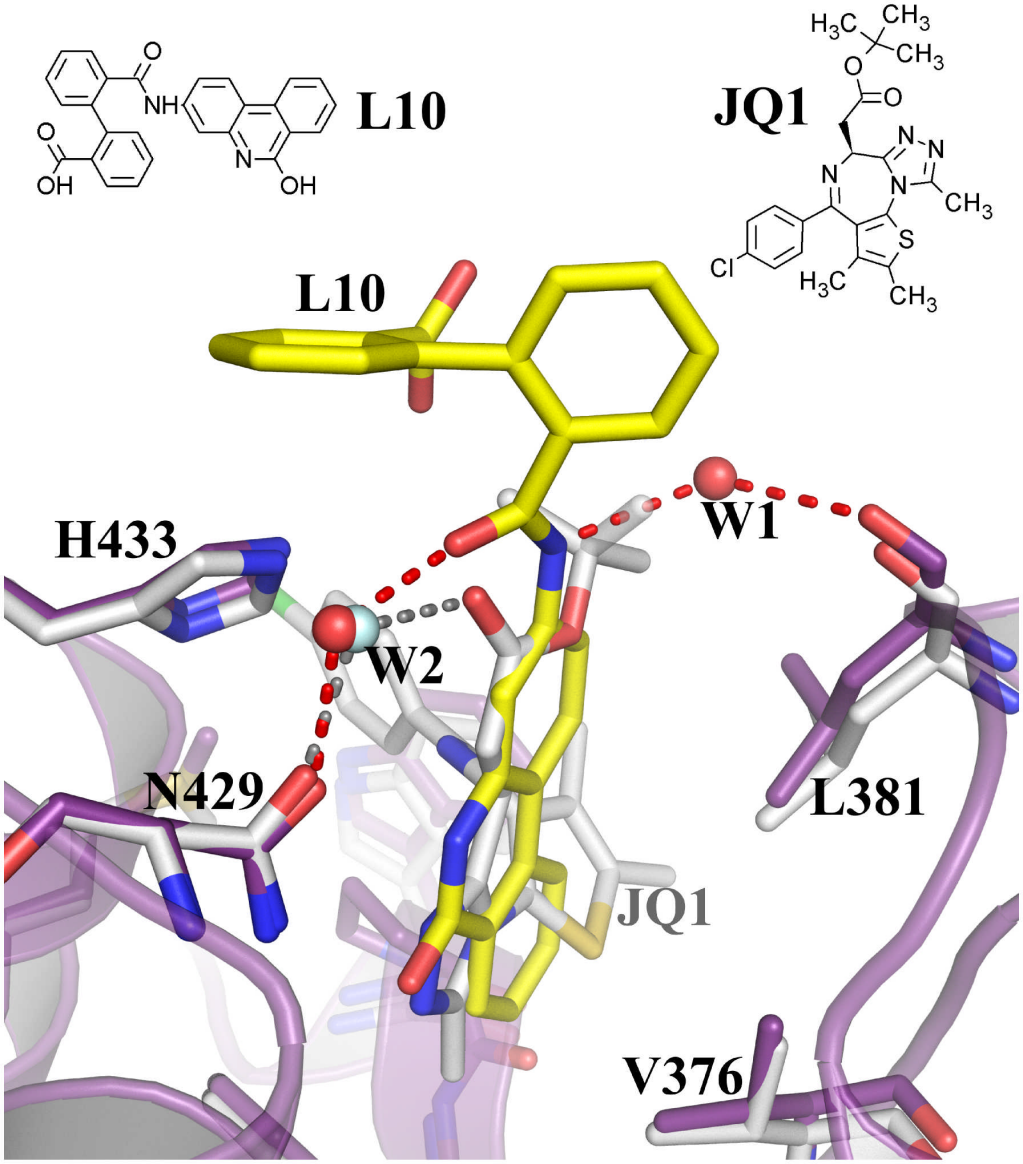
Comparison of water bridged interactions formed in the JQ1 and L10 complexes at the edge of the binding pocket. Two water molecules (W1 and W2) are demonstrating water bridged hydrogen bonds (red) in the *BD2-L10* complex. A single water mediated hydrogen bond is observed in the case of JQ1 (grey). Compound L10 is shown in yellow. The residues corresponding to *BD2-L10* and *BD2-JQ1* are shown in purple and grey, respectively. The ligands and the interacting residues are shown as sticks. Water molecules are shown as spheres. The hydrogen bonds are shown as broken lines.

To compare the L10 complex with JQ1 complex, we superposed the two structures onto each other (Fig 8). The WPF shelf region is occupied by a part of JQ1 molecule (chlorophenyl moiety) which is absent in L10 complex (S4 Fig). Intriguingly, a similar mode of two water-mediated intermolecular hydrogen bonds with N429 and H433 are observed in both complexes (Fig 8). It suggests that this type of interactions may be essential to stabilize a compound at the edge of the binding pocket region.

### Weak interactions and speculated modifications in L10

The compound L10 displays overall good hydrogen bonding interactions with the otherwise mostly hydrophobic binding site of BD2. On visualizing the electron density maps, one can clearly observe a very well defined electron density profile of the phenanthridinone moiety, but a poor electron density profile of the terminal phenyl-benzoic acid group, which are present just outside the Kac binding pocket (Fig 1B). It clearly suggests that the lack of suitable intermolecular interactions and relatively large size of the terminal phenyl-benzoic acid group of L10 is the possible reason for the moderate affinity of L10 towards BD2. In order to stabilize this part of L10 on the edge of the BD2 binding site, modifications would be necessary for this portion of L10. The main modification can be the complete removal of the terminal ring and replace it with a–OH or a–OCH_3_ group, and/or an additional–OH or–OCH_3_ group attached to a C18 atom. This can help in forming new hydrogen bond (towards H433 NE2) and hydrophobic interactions (towards P430), on the edge of the cavity, and can improve the overall binding of L10 with BD2. A structural feature which makes JQ1 and RVX-208 superior compared to L10, is the presence of hydrophobic groups like chlorophenyl and thienol (in JQ1) and methoxide groups (in RVX-208). We also speculate that the presence of a methoxide group on the phenanthridinone group, towards the ZA loop side of L10 (similar to RVX-208), may help improve its interactions within the binding pocket. It may also help in forming new hydrogen bonding interactions between the methoxide oxygen and conserved water molecules, and hydrophobic interactions between the methyl group of such a substituent to W370 and P371. These modifications can help in increasing overall interactions, and thus, improve the overall stability of the compound in the BD2 binding pocket.

## Conclusion

The high resolution crystal structure enabled us to identify new structural features of BD2 bromodomain. A novel phenanthridinone based ligand has been identified, showing significant interactions in the BD2 binding pocket, which are comparable to the previously known inhibitors. Weaker electron density profile of terminal phenyl-benzoic acid rings indicates a lack of suitable interactions of this portion of L10 towards BD2. Chemical modifications of L10 in the terminal phenyl-benzoic acid ring portion may help improve the binding of L10 in the BD2 binding pocket. The ligand can serve as an excellent template, to identify novel phenanthridinone based BD2 inhibitors.

## Supporting Information

S1 Fig Validation study for molecular docking by PyRx for the known *BD2-JQ1* complex. The crystal (yellow; PDB Id: 3ONI) and docked structures (cyan) of the JQ1 compound, are shown as sticks.
(TIF)

S2 Fig Superposition of docked L10 structure compared to the one observed in crystals. The crystal (green) and docked structures (cyan) of the L10 compound are shown as sticks.
(TIF)

S3 Fig Alternate conformations of residues in the *apo-*form of BD2. **(A)** Alternative conformations are demonstrated by residues C425 and N424, present near the conserved water molecules in the binding pocket. **B.** Alternative conformations demonstrated by F422 and Q443. The representative residues, which possess alternate conformations, are shown as sticks. Water molecules are shown as spheres. **|**2Fo|-|Fc| map of representative residues contoured at 1.0σ.
(TIF)

S4 Fig Superposition of the structures of *BD2-JQ1* and *BD2-L10* complex. JQ1 and L10 are represented in grey and yellow, respectively. The interacting residues in the binding site region and the ligands are shown as sticks. Residues corresponding to the *BD2-L10* complex are shown in purple.
(TIF)

S1 Table Docking energy analysis of top hits obtained from docking of BRD2-BD2 with NCI diversity III. (DOCX)

S2 Table RMSD values calculated between C^α^ atoms of whole protein and active site residues (ZA loop: residues 370–384, BC loop: residues 421–435) comparing complexes *BD2-L10*, *apo-BD2*, *BD2-RVX-208*, and *BD2-JQ1*. (DOCX)
