# Supplementary figures and images for: A Novel Phenanthridionone Based Scaffold As a Potential Inhibitor of the BRD2 Bromodomain: Crystal Structure of the Complex
Source: PLoS One. 2016 May 31;11(5):e0156344. doi: 10.1371/journal.pone.0156344 (PMC4886958; doi:10.1371/journal.pone.0156344)

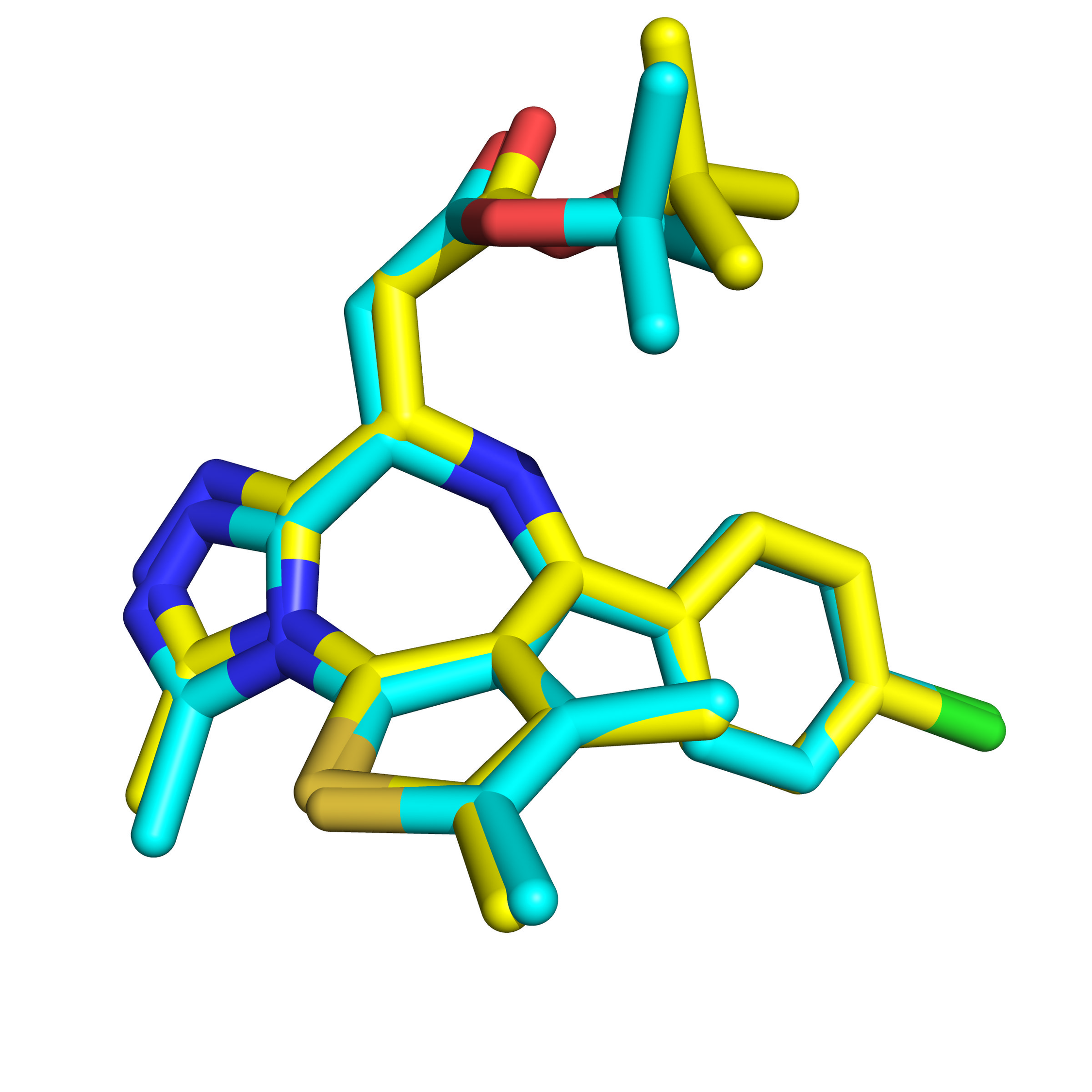

Supplement: S1 Fig — The crystal (yellow; PDB Id: 3ONI) and docked structures (cyan) of the JQ1 compound, are shown as sticks. (TIF) [file pone.0156344.s001.tif]

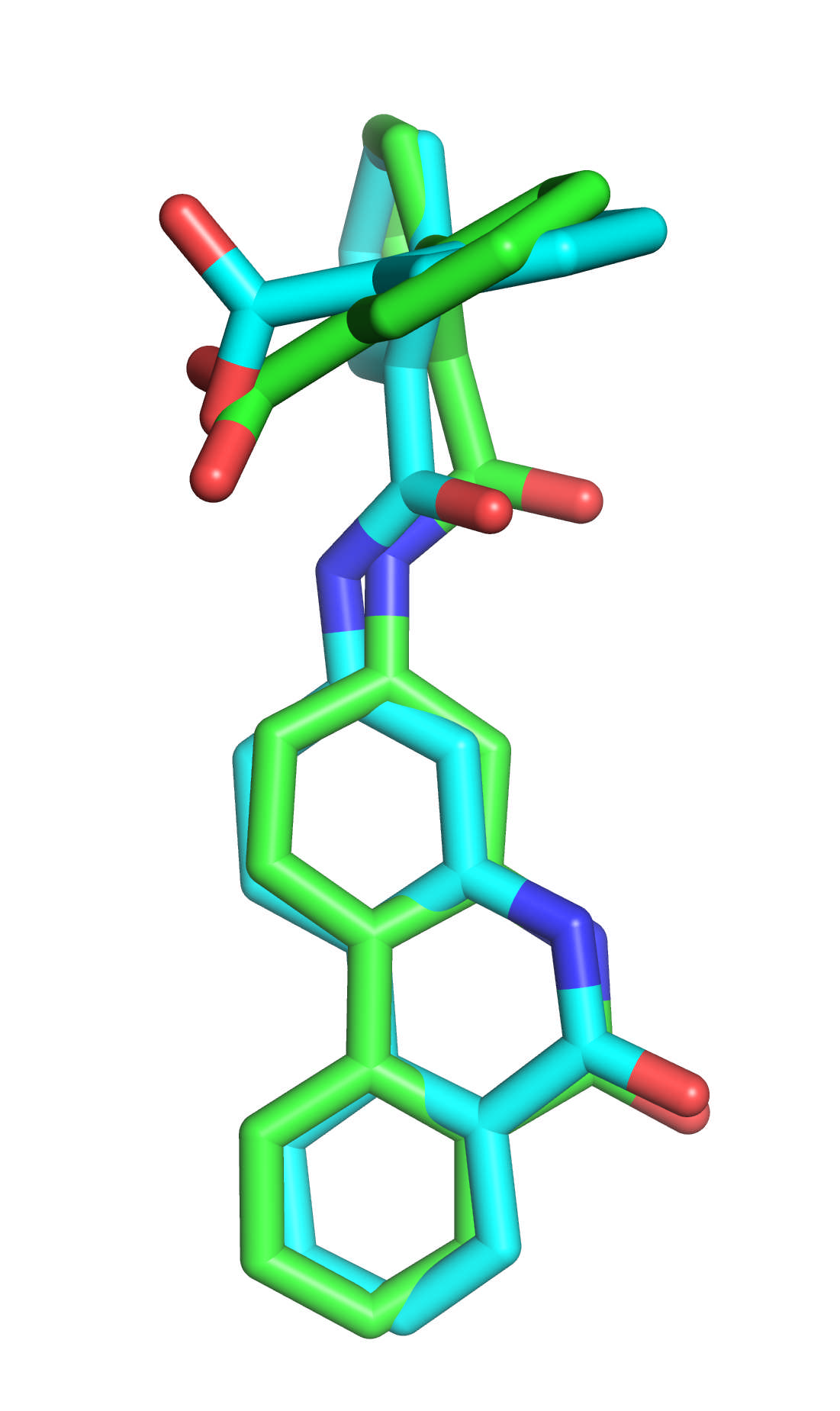

Supplement: S2 Fig — The crystal (green) and docked structures (cyan) of the L10 compound are shown as sticks. (TIF) [file pone.0156344.s002.tif]

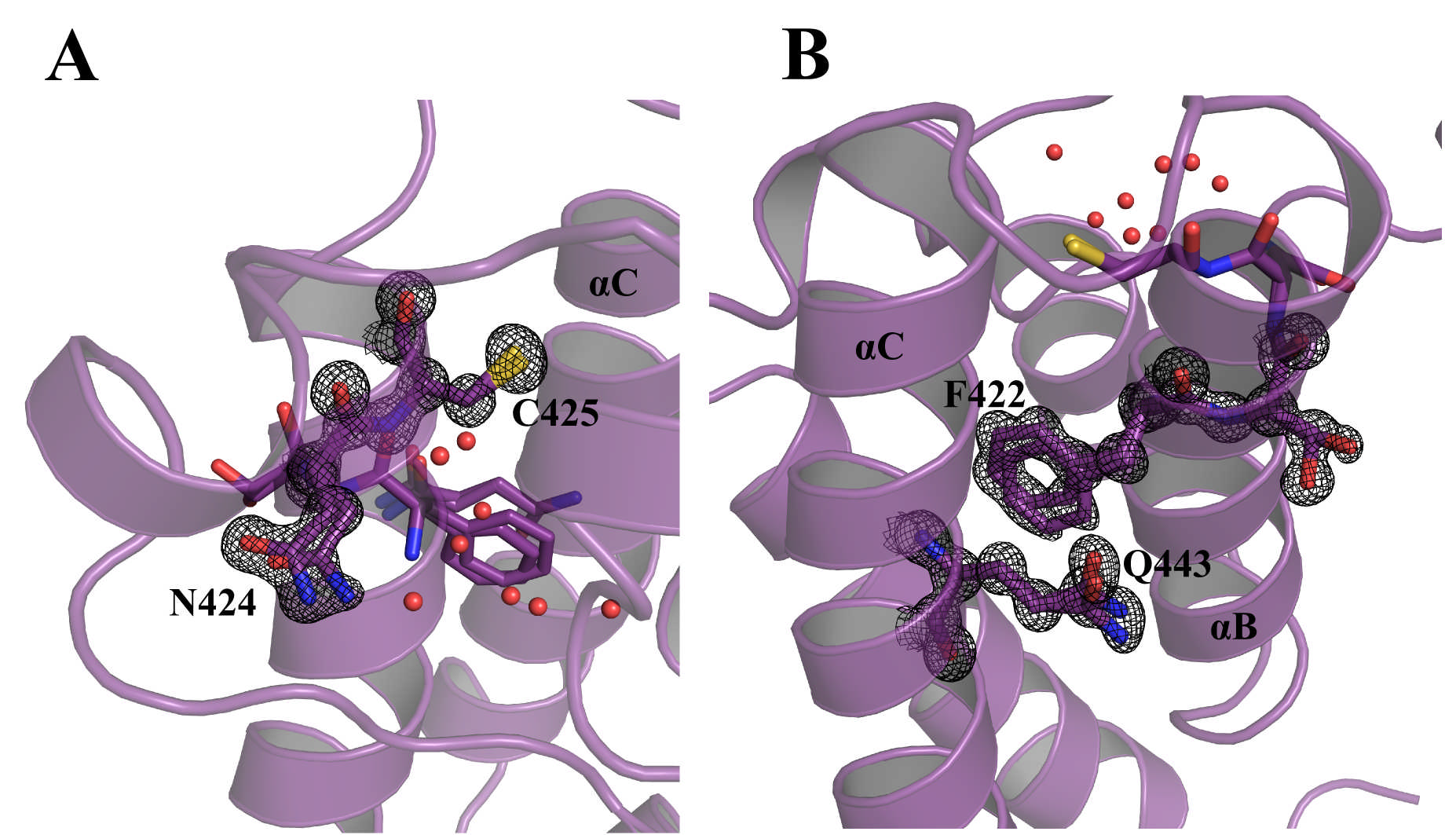

Supplement: S3 Fig — (A) Alternative conformations are demonstrated by residues C425 and N424, present near the conserved water molecules in the binding pocket. B. Alternative conformations demonstrated by F422 and Q443. The representative residues, which possess alternate conformations, are shown as sticks. Water molecules are shown as spheres. |2Fo|-|Fc| map of representative residues contoured at 1.0σ. (TIF) [file pone.0156344.s003.tif]

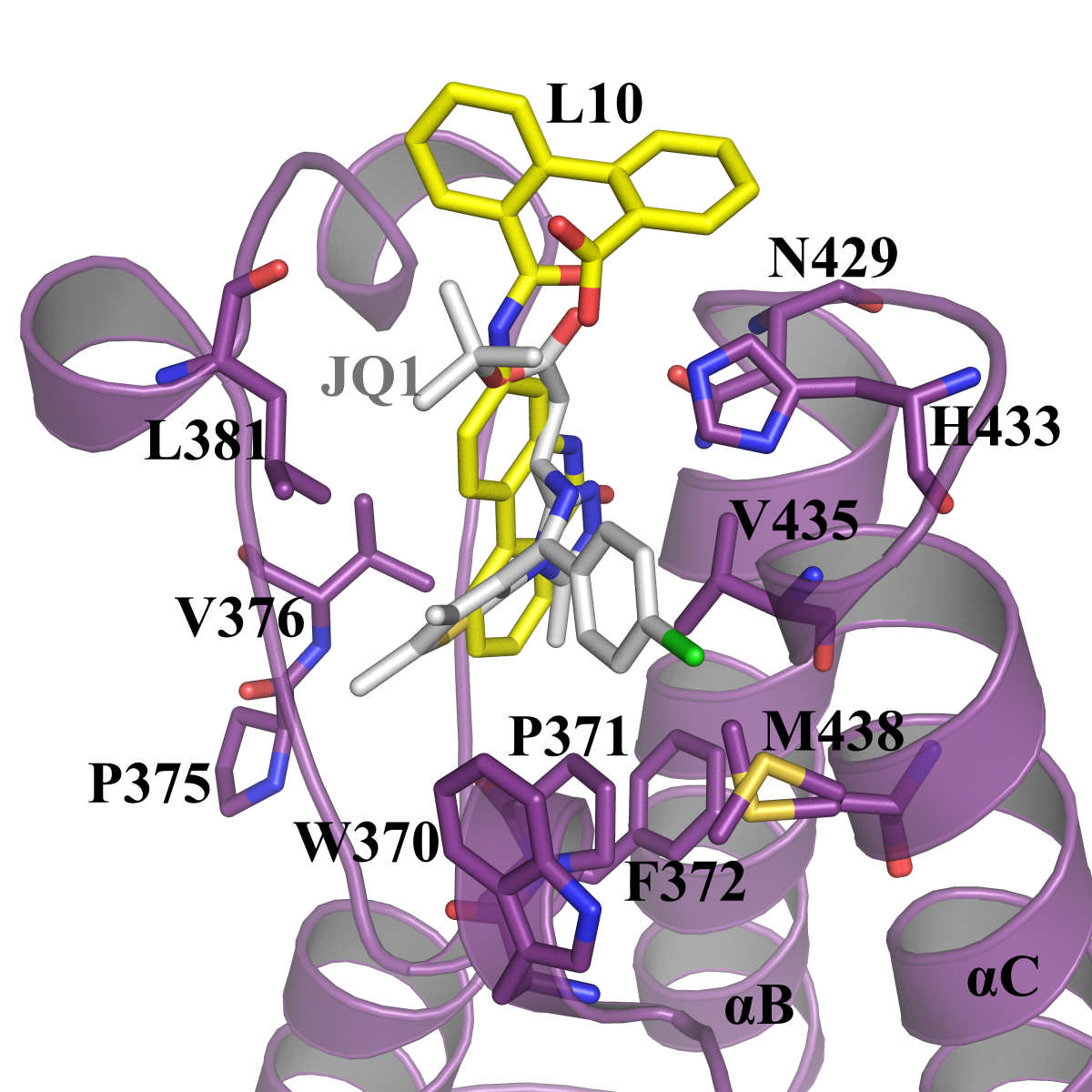

Supplement: S4 Fig — JQ1 and L10 are represented in grey and yellow, respectively. The interacting residues in the binding site region and the ligands are shown as sticks. Residues corresponding to the BD2-L10 complex are shown in purple. (TIF) [file pone.0156344.s004.tif]
