# Supplementary material for: A Novel Phenanthridionone Based Scaffold As a Potential Inhibitor of the BRD2 Bromodomain: Crystal Structure of the Complex
Source: PLoS One. 2016 May 31;11(5):e0156344. doi: 10.1371/journal.pone.0156344 (PMC4886958; doi:10.1371/journal.pone.0156344)
Supplement: S1 Table — (DOCX) [file pone.0156344.s005.docx]

**S1 Table.** Docking energy analysis of top hits obtained from docking of BRD2(BD2) with NCI diversity III.

| **S.no** | **Compound code** | **Compound name** | 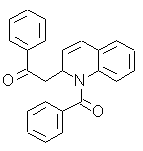**Structure** | **Binding affinity (kcal/mol)** | **Ligand efficiency (kcal/mol per non hydrogen atom)** |
| --- | --- | --- | --- | --- | --- |
| 1 | 835 | 1,6,5. 2-(1-benzoyl-2H-quinolin-2-yl)-1-phenylethanone |  | -9.9 | -0.3667 |
| 2 | 1013 | 1,8-bis(phenylsulfanyl)anthracene-9,10-dione | 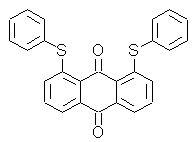 | -9.7 | -0.3233 |
| 3. | 518 | 12a,14a-dimethyl-1,2,3,3a,3b,4,5,5a,6,7,12,12a,12b,13,14,14a-hexadecahydrocyclopenta[5,6]naphtho[2,1-b]carbazol-1-o | 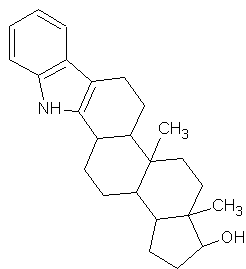 | -9.6 | -0.3556 |
| 4. | 834 | 1-(4-(4-methylphenyl)-5-phenyl-1,3-oxazol-2-yl)isoquinoline | 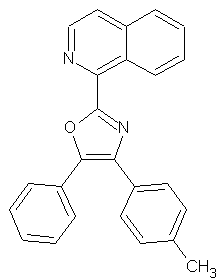 | -9.6 | -0.3429 |
| 5. | 932 | 2-nitro-3-phenylspiro[cyclopropane-1,9'-fluorene] | 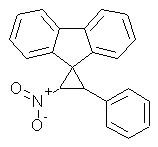 | -9.3 | -0.3875 |
| 6 | 116 | 4-[(6-chloro-2-methoxyacridin-9-yl)amino]-5-methyl-2-propan-2-ylphenol | 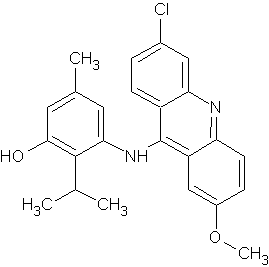 | -9.2 | -0.3172 |
| 7 | 1041 | 2-(5,11-dimethyl-6H-pyrido[4,3-b]carbazol-6-yl)ethyl benzoate | 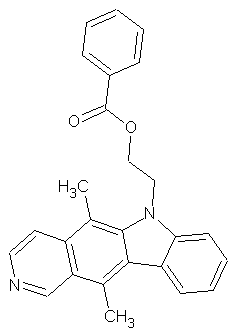 | -9.1 | -0.3033 |
| 8 | 1395 | 1,6,5. 2-(1-benzoyl-2H-quinolin-2-yl)-1-phenylethanone | 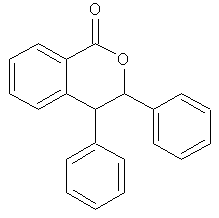 | -9.1 | -0.3792 |
| 9 | 477 | 1,8-bis(phenylsulfanyl)anthracene-9,10-dione | 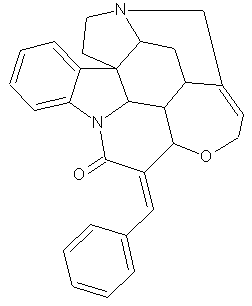 | -9.7 | -0.3957 |
| 10 | 865 | 2-[2-[(6-oxo-5H-phenanthridin-3-yl)carbamoyl]phenyl]benzoic acid | 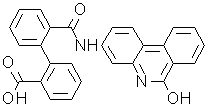 | -9.1 | -0.2844 |
