# Supplementary material for: A Novel Phenanthridionone Based Scaffold As a Potential Inhibitor of the BRD2 Bromodomain: Crystal Structure of the Complex
Source: PLoS One. 2016 May 31;11(5):e0156344. doi: 10.1371/journal.pone.0156344 (PMC4886958; doi:10.1371/journal.pone.0156344)
Supplement: S2 Table — (DOCX) [file pone.0156344.s006.docx]

**S2 Table.** RMSD values calculated between C^α^ atoms of whole protein and active site residues (ZA loop: residues 370-384, BC loop: residues 421-435) comparing complexes BD2-L10, apo-BD2, BD2-RVX-208, and BD2-JQ1.

| **Superposition between the structures** | **RMSD (Å) for all C^α^ atoms (103 atoms).** | **RMSD (Å) for the C^α^ atoms in the binding site region covering ZA and BC loops (32 atoms).** |
| --- | --- | --- |
| BD2-L10 and apo-BD2 | 0.078 | 0.087 |
| BD2-L10 and JQ1-BD2 | 0.080 | 0.078 |
| BD2-L10 and BD2-RVX-208 | 0.301 | 0.273 |
| apo-BD2 and BD2-JQ1 | 0.099 | 0.184 |
| apo-BD2 and BD2- RVX-208 | 0.342 | 0.381 |
| BD2-JQ1 and BD2- RVX-208 | 0.261 | 0.252 |
